# Influence of Microbial Transglutaminase on the Formation of Physico-Chemical Properties of Meat Analogs

**DOI:** 10.3390/foods13244085

**Published:** 2024-12-17

**Authors:** Anna Zimoch-Korzycka, Anna Krawczyk, Żaneta Król-Kilińska, Dominika Kulig, Łukasz Bobak, Andrzej Jarmoluk

**Affiliations:** Department of Functional Food Product Development, Wroclaw University of Environmental and Life Sciences, 37 Chelmonskiego Str., 51-630 Wroclaw, Poland; anna.zimoch-korzycka@upwr.edu.pl (A.Z.-K.); zaneta.krol@upwr.edu.pl (Ż.K.-K.); dominika.kulig@upwr.edu.pl (D.K.); lukasz.bobak@upwr.edu.pl (Ł.B.); andrzej.jarmoluk@upwr.edu.pl (A.J.)

**Keywords:** microbial transglutaminase, incubation time, protein cross-linking, texture profile, physico-chemical parameters, chicken meat sausage analog

## Abstract

With growing environmental and health concerns surrounding meat consumption, meat analogs have emerged as sustainable and health-conscious alternatives. A major challenge in developing these products is replicating the fibrous, elastic texture of meat, where microbial transglutaminase (MTG) has shown significant potential. MTG catalyzes protein cross-linking, enhancing the structural integrity of meat analogs. This study aimed to evaluate the effects of MTG concentrations (0%, 0.5%, and 1%) and incubation times (0, 1.5, and 3 h) on the quality and rheological properties of meat analogs. Analogs were tested for pH, protein content, dry matter, fat retention, and thermal loss. Textural properties, including hardness, cohesiveness, gumminess, springiness, and chewiness, were determined using texture profile analysis, while leakage parameters were evaluated through water and fat content tests. Results revealed that higher MTG concentrations and longer incubation times improved protein content (14.34% to 15.55%), dry matter (29.61% to 32.53%), and reduced total leakage (1.262% to 0.634%). Textural properties, including hardness (57.08 N to 83.14 N), gumminess (19.40 N to 30.00 N), and chewiness (17.60 N × mm to 29.58 N × mm), also significantly improved with increasing MTG levels. Thermal loss ranged from 98.37% to 100.9%, showing enhanced retention at higher MTG concentrations. These results support the role of MTG in creating meat analogs with improved meat-like textures, achieved through enhanced protein cross-linking and moisture retention.

## 1. Introduction

For an extended period, meat has been regarded as a key component of the human diet due to its complete profile of essential amino acids and the high digestibility of its protein [1,2]. Since 1961, global meat consumption has nearly doubled, increasing from approximately 23 kg per person per year to around 43 kg by 2021 [3]. This significant increase can be attributed to population growth, urbanization, and evolving dietary preferences, particularly in developing countries where rising incomes have led to increased meat consumption [4]. In contrast, while developed countries remain substantial consumers, they have experienced a more moderate increase, reflecting changes in dietary patterns [5,6]. A key factor driving this shift is the growing concern about the adverse effects of excessive meat consumption on both health and the environment.

Reducing meat consumption in the diet is associated with numerous health benefits, contributing to overall well-being and reducing the risk of chronic diseases [7]. Many research findings indicate that a decrease in meat intake, particularly red and processed varieties, is correlated with a reduced risk of cardiovascular disease, type 2 diabetes, and certain cancers, especially colon cancer [8,9,10]. There is evidence that decreasing meat consumption can lead to significant environmental benefits, including lower greenhouse gas emissions, less water consumption, and less land degradation. Studies suggest that even a slight reduction in meat intake can lead to a significant decrease in resource consumption and environmental impact [11,12,13].

As consumers become increasingly aware of their dietary choices, many are deciding to reduce their meat consumption or seek alternative protein sources that align with their health and ethical considerations [14,15]. Consequently, the growing demand for meat analogs has led to the emergence of innovative food technologies aimed at meeting consumer preferences, not only in terms of nutritional value but also with regard to sensory characteristics [16,17].

Despite the increasing popularity of meat analogs, major challenges remain in optimizing production processes. A primary obstacle in developing meat analogs is achieving a texture that closely resembles the fibrous, elastic, and juicy characteristics of an animal muscle [18]. Traditionally, high-moisture extrusion has been employed, applying heat and shear forces to a protein mixture, aligning the proteins into a fibrous structure that partially mimics meat. However, while extrusion can produce meat-like textures, it has notable limitations. This process requires high energy and precise temperature control to prevent protein degradation [19]. Furthermore, the extrusion process can impart undesirable off-flavors and odors to the final product, which may be unappealing to consumers looking for a close replication of meat’s similar profile [20]. To overcome these challenges, researchers are exploring alternative methods to enhance the physical and functional qualities of meat analogs. A promising approach involves the incorporation of hydrocolloids and enzymatic preparations into the meat analog formulations [21].

Incorporation of hydrocolloids, such as alginate and methylcellulose, into meat analogs presents a promising alternative to extrusion, offering flexibility in texture formation. Hydrocolloids are well-known for their ability to form gels and modify the texture and moisture retention properties of food products [22]. When combined with proteins, hydrocolloids can create hydrocolloid-protein biocomposites that mimic the microstructural characteristics of meat [23]. When a solution containing a protein isolate and sodium alginate is injected into a calcium chloride solution, gelation occurs due to calcium ions cross-linking alginate molecules. This results in the formation of fibers that closely resemble the structure of meat fibers while maintaining a high protein content [24]. Proteins participate in creating the external appearance, color, juiciness, and texture of food due to its functional properties including gelation, solubility, water holding capacity, emulsification, and nutrition value [25].

Transglutaminase is an enzyme that occurs naturally in various organisms. Initially, this enzyme was obtained from guinea pig liver, fish tissue, and plant tissue. It was soon discovered in microorganisms grown in appropriate media synthesize extracellular transglutaminase, which is then isolated using methods such as ultrafiltration, evaporative concentration, or salting out with inorganic salts. Unlike animal transglutaminase, microbial transglutaminase is calcium-independent, has a smaller molecular weight, and is much cheaper to produce [26]. The Food and Drug Administration approved the use of microbial transglutaminase as a “Generally Recognized as Safe—GRAS” for food processing in 1998. Microbial transglutaminase is considered safe, nontoxic, nonallergenic, nonimmunogenic, and non-pathogenic for public health. Additionally, there is no requirement to list it among the products’ ingredients [27].

Microbial transglutaminase plays a key role in the formation of meat analogs by catalyzing protein cross-linking, specifically through transamidation. This reaction involves the formation of covalent bonds between proteins by linking amino acid residues of glutamine and lysine. This process can occur both intramolecularly (within a single protein molecule) and intermolecularly (between different protein molecules), leading to the formation of protein polymers [28]. By facilitating this process, microbial transglutaminase strengthens the protein matrix, imparting elasticity and firmness to the product [29]. This combination not only stabilizes the fibrous structure formed during ionic gelation but also provides a texture that provides a better imitation of the chewiness and elasticity of meat fibers. Enzymatic treatment improves texture attributes such as hardness and cohesiveness, which are critical for consumer acceptance of meat analogs [30].

The advantages of using protein–hydrocolloid composites cross-linked by microbial transglutaminase lie in the lower energy input compared to the extrusion process and the fact that they do not rely on high temperatures, preserving the integrity and nutritional quality of the proteins. Furthermore, these composites allow for improved control over mechanical strength by adjusting microbial transglutaminase concentration and incubation time [31]. Consequently, the main objective of this study was to propose a protein texturization process using microbial transglutaminase to produce a meat-like texture and structure by investigating the effects of varying concentrations of microbial transglutaminase and different incubation times on the rheological properties of composite materials, along with an assessment of their quality parameters.

## 2. Materials and Methods

### 2.1. Materials

Egg protein albumin was obtained from Ovopol Sp. z o.o., while acid casein (85.7% proteins) was produced by Polsero Sp. z o. o. (Sokołów Podlaski, Poland). Microbial transglutaminase, derived from the culture of *Streptoverticillium* spp.—MTG, ACTIVA WM (99% maltodextrine and 1% microbial transglutaminase; activity of ≈96 U/g) was purchased from Ajinomoto Foods Europe SAS (Paris, France). Sodium alginate FD 125 and methylcellulose (Methocel™) were procured from Dupont GRINSTED^®^ (Grindsted, Denmark and Dow Wolff Cellulosics Gmbh (Bomlitz, Germany), respectively. The commercially available wheat fiber WF 300 and pea fiber EF 150 were sourced from Vitacel, Rettenmaier (Warsaw, Poland). Anhydrous calcium chloride was purchased from the company P.P.H. “STANLAB” s.j. (Lublin, Poland) and canola oil was obtained from a local supermarket (Tesco, Wrocław, Poland). Flavor additives and colorants used included cochineal red E124 and brown HT E155 (Food Colours Perczak S.J., Piotrkow Trybunalski, Poland), sodium chloride, ground black pepper, herbal pepper, ground white pepper (McCormick Polska S.A., Stefanowo, Poland), a chicken-vegetable bouillon cube (Prymat Sp. z o.o., Jastrzebie Zdroj, Poland), and sausage flavoring (ŻUK—POL Sp. Z o. o., Wroclaw, Poland).

### 2.2. Methods

#### 2.2.1. Samples Preparation

The process of preparing chicken meat sausage analog began with the formulation of the protein solution in a 250 mL beaker. The combination of 68.5% water and 7.5% canola oil was heated to 70 °C using a water bath (TW 12 series, JULABO GmbH, Seelbach, Germany) and stirred with a R50 CAT mechanical stirrer (Ballrechten-Dottingen, Germany) fitted with a propeller blade at 350 rpm. Additional ingredients were added gradually, increasing the stirring speed to 600 rpm. First, 8% acid casein was stirred for 10 min, then 1.9% sodium alginate was stirred for 15 min, followed by 0.3% methyl cellulose for 3 min, and finally 2.7% flavoring and 2% coloring was added.

The development of protein–hydrocolloidal fibers involved the addition of anhydrous calcium chloride (0.6%) to a protein solution, which was kept at 70 °C. A mechanical stirrer and a flat-ended spatula were used to mix the solution at 600 rpm, preventing fiber clumping and ensuring a consistent blend. Afterward, the mixture was allowed to cool to room temperature, at which point the remaining ingredients were added manually. Albumin was incorporated at a concentration of 6%, followed by the addition of fibers—WF300 and EF150—each at 1%, and MTG at varying concentrations of 0, 0.5, and 1 (in powder preparate) depending on the specific variant presented in Table 1.

**Figure 1:**
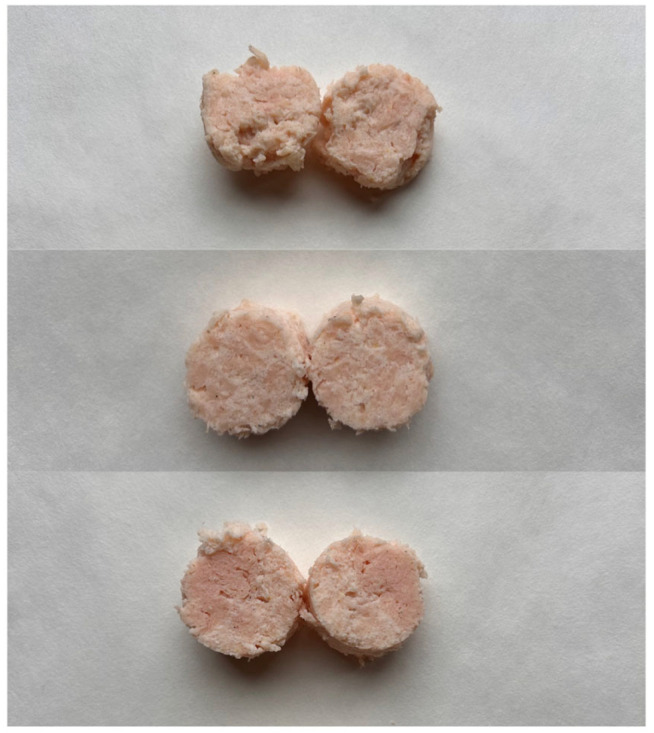
Samples after 1.5 h of incubation from the top with 0, 0.5 and 1% of MTG, respectively.

The blend was subsequently sealed in 23 mm diameter cellulose casings (Lommel Belgium) using a stuffing machine and secured with butcher’s string. The samples were then incubated in an incubator (UNE 400, Memmert, Schwabach, Germany) at 55 °C for 90 or 180 min or left unincubated. After this incubation period, the samples were pasteurized to reach an internal temperature of 70 °C, cooled to ambient temperature in cold water, stored in the refrigerator for 24 h and then re-warmed to room temperature prior to analysis (Figure 1).

#### 2.2.2. pH Determination

The measurement of acidity was performed for each sample after 24 h of refrigeration, using a pH meter (inoLab, Weilheim, Germany). Prior to analysis, the device was calibrated using buffer solutions (pH 4 and 7). The pH value was obtained by immersing a calomel electrode in the prepared homogenates.

#### 2.2.3. Protein Content Determination

The total nitrogen content was determined using the Kjeldahl method with the Kjeltec 2300 Analyzer (FOSS, Hillerod, Denmark), in accordance with the standard PN-75/A-04018 [32]. This method involves the conversion of organic nitrogen compounds into (NH_4_)_2_SO_4_ through the application of concentrated H_2_SO_4_ in the presence of a catalyst K_2_SO_4_ and CuSO_4_ × 5H_2_O. Subsequently, the solution was alkalized, distilled, and titrated with a 0.1 N HCl solution until a pink-violet color was achieved. The protein content was calculated using a universal nitrogen conversion factor of 6.25.

#### 2.2.4. Determination of Dry Matter

This determination was performed based on the Polish Standard PN-ISO 1442:2000 [33], utilizing a thermal drying method. Samples of 3 g of the ground material were prepared and weighed with an accuracy of 0.001 g. The samples were dried at a temperature of 103 ± 2 °C until a constant weight was achieved. The samples were then cooled in a desiccator until they reached room temperature. Then, the samples were weighed again with an accuracy of 0.001 g. The difference in mass of the samples before and after drying represented the dry matter content. Calculations were performed using the following formula:

(1) $$\%DM=\frac{a\;}{b}\times 100$$

where: *DM*—dry matter [%]; *a*—mass of the sample after drying [g]; *b*—mass of the sample before drying [g].

#### 2.2.5. Determination of Fat Content and Dry Matter of Defatted Mass

The determination was conducted according to the Polish Standard PN-ISO 1444:2000 [34] using an extraction method with a Soxhlet apparatus. The principle of the method is based on the extraction of a dried sample using petroleum ether. The solvent residue was removed through evaporation and drying, ensuring that the samples reached a constant weight. The difference in mass before and after extraction represented the fat content. To calculate the fat content, the following formula was employed:

(2) $$\%FC=\frac{M-m}{n}\times 100$$

where *FC*—free fat content in the product [%]; *M*—mass of the filter paper with the fat sample after drying [g]; *m*—mass of the filter paper after extraction and drying [g]; *n*—mass of the fat sample before drying [g].

For the calculation of the dry matter of defatted mass, the formula used was

(3) %DMDF=%DM−%FC

where *DMDF*—dry matter of defatted mass [%]; *DM*—dry matter [%]; *FC*—free fat content in the product [%].

#### 2.2.6. Identification of the Total Water and Fat Leakage, the Water and the Fat Content in the Total Leakage

The determination was performed according to the method established by Lee and Patel [35]. Samples measuring 15 mm in height were cut from cylindrical blocks with a diameter of 20 mm. Each sample was placed between two pre-weighed Grade 1 (Whatman, Maidston, UK) filter papers, with a precision of 0.001 g, and subsequently compressed to a 40% deformation relative to the initial height of the sample using the Z010 testing device (Zwick Roell, Ulm, Germany). When the specified deformation level was reached, compression was halted, and the sample was kept under load for 60 s. Subsequently, the compressing head was retracted to its original position, and the samples were removed from between the papers, which were then weighed again with a precision of 0.001 g. The weighed papers were dried at a temperature of 103 ± 2 °C until a constant mass was achieved.

Based on the measurements obtained, the total water and fat leakage of the sample (TL), the water content in the total leakage (WL), and the fat content in the total leakage (FL) were calculated. The formula used to determine the total water and fat leakage is as follows:

(4) $$\%TL=\frac{m_{pa}-m_{pb}}{M}\times 100$$

where *TL* represents the total water and fat leakage from the sample [%]; *m_pa_* denotes the mass of the paper after compression [g]; *m_pb_* indicates the mass of the paper before compression [g]; *M* represents the mass of the sample [g].

For calculating the water content in the total leakage, the formula used is

(5) $$\%WL=\frac{m_{pa}-m_{pd}}{M}\times 100$$

where *WL* indicates the water content in the total leakage [%]; *m_pa_* is the mass of the paper after compression [g]; *m_pd_* is the mass of the paper after drying [g]; *M* denotes the mass of the sample [g].

To calculate the fat content in the total leakage, the following formula was employed:

(6) %FL=%TL−%WL

where *FL* denotes the fat content in the total leakage [%]; *TL* represents the total water and fat leakage from the sample [%]; *WL* indicates the water content in the total leakage [%].

#### 2.2.7. Thermal Loss Determination

To assess the mass loss resulting from thermal processing, samples were weighed both prior to and after pasteurization. Losses were calculated as the difference in mass of the samples before and after thermal treatment. The results were presented as the efficiency of the process.

#### 2.2.8. Determination of Selected Textural Parameters

The determination of the texture profile and selected viscoelastic parameters was performed using the Z010 testing device (Zwick Roell, Ulm, Germany). Samples were analyzed after being stored for 24 h under refrigeration, following a prior heating to room temperature. Cylindrical samples (15 mm × 23 mm, H × d) were cut from the bars. These samples underwent a 75% deformation test through double compression (TPA) and included a relaxation time of 30 s between compressions. For the TPA test, five rheological parameters were quantified: hardness [N] was defined as the maximum force applied to the sample during initial compression. Springiness [-] indicated the distance traveled by the probe during the second compression cycle. Cohesiveness [-] was the ratio of work undertaken in the second compression to that in the first. Gumminess [N] was calculated as the product of hardness and cohesiveness, while chewiness [N × mm] was determined as the product of gumminess and springiness [36].

### 2.3. Statistical Analysis

Statistical analysis of the results was performed using GraphPad Prism software, version 10.3.1 (464) (GraphPad Software, San Diego, CA, USA). A two-way analysis of variance (ANOVA) was applied to process the data, and the significance of differences between groups was evaluated using Tukey’s post hoc test, with the significance level set at 0.05. All evaluations were performed in triplicate unless mentioned otherwise.

## 3. Results and Discussion

### 3.1. pH

**Figure 2:**
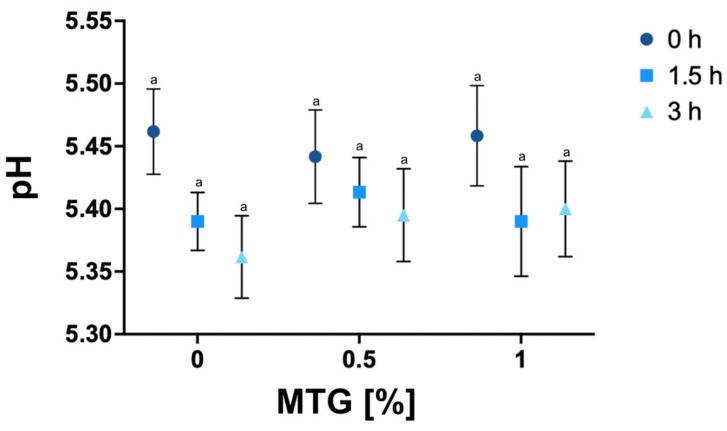
pH values of meat analogs with MTG concentrations of 0%, 0.5%, and 1% over incubation times of 0, 1.5, and 3 h. The data represent the mean and the error bars represent the standard deviation (*n* = 6). ^a^ mean statistically significant differences (*p* ≤ 0.05).

The pH results of of all tested variants are shown in Figure 2. The lowest mean pH value was recorded for the 0_MTG_3T variant (5.36), while the highest pH was observed in the 0_MTG_0T variant (5.46). This relatively narrow range in pH values indicates a notable stability across the different formulations, suggesting that neither MTG concentration nor incubation time significantly affects the pH levels of meat analogs. Such findings are consistent with previous studies reporting similar pH stability during enzymatic protein structure modification, undersoring the resilience of non-meat protein analog formulations to changes in processing parameters without significant shifts in pH [37].

### 3.2. Protein Content

**Figure 3:**
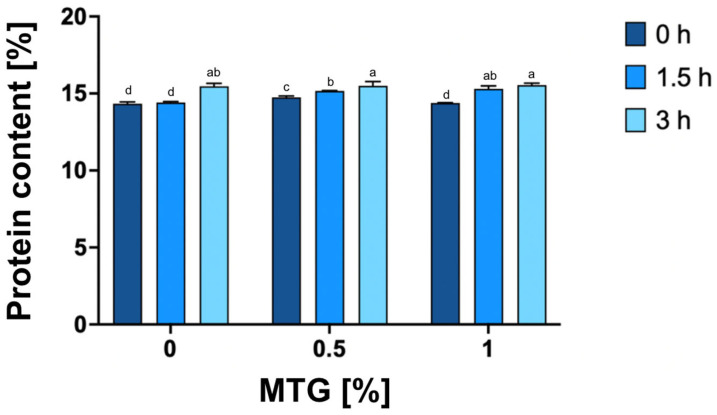
Protein content [%] of meat analogs cross-linked with different concentrations of MTG (0%, 0.5%, and 1%) over three incubation times (0 h, 1.5 h, and 3 h). The data represent the mean and the error bars represent the standard deviation (*n* = 5). ^a–d^ means statistically significant differences (*p* ≤ 0.05).

The study revealed significant effects of both MTG concentration and incubation time on protein content (*p* < 0.0001), as well as a notable interaction between these factors (*p* < 0.0001), indicating their combined influence on the protein levels in the samples (Figure 3). The highest protein content was observed in the 1_MTG_3T variant (15.55), while the lowest was recorded in the 0_MTG_0T, with a mean value of 14.34. The intermediate values observed in the 0_MTG_3T variant suggest that prolonged incubation alone may induce minor protein interactions through non-enzymatic processes; however, the presence of MTG significantly amplifies these effects. These findings underscore a significant relationship between increased concentrations of microbial transglutaminase and extended incubation times, which likely enhance protein content through cumulative cross-linking effects induced by MTG over time. This aligns with previous research indicating that enzyme concentration and incubation duration are pivotal for optimizing protein properties in food systems, as higher enzyme levels promote more extensive protein network formation and stabilization through cross-linking mechanisms [38,39,40].

### 3.3. Dry Matter, Fat Content, and Dry Matter of Defatted Mass

**Figure 4:**
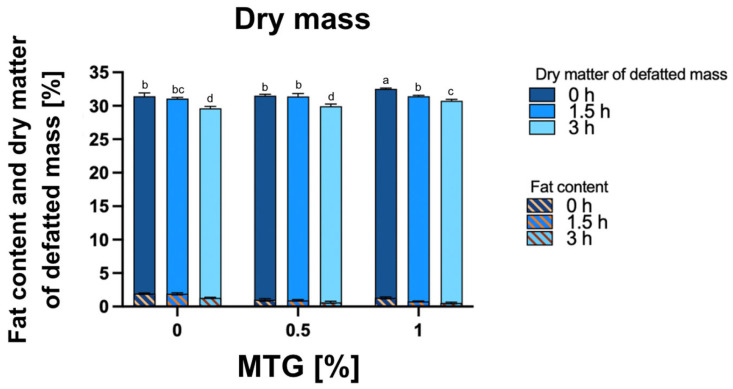
Dry mass, fat content, and dry matter of defatted mass [%] in meat analogs with varying MTG concentrations (0%, 0.5%, 1%) across incubation times (0 h, 1.5 h, and 3 h). The data represent the mean and the error bars represent the standard deviation (*n* = 6). ^a–d^ means statistically significant differences (*p* ≤ 0.05).

For dry matter, the interaction between MTG concentration and incubation time accounted for a significant portion of the total variation (*p* = 0.0017), indicating that the effects of MTG concentration and incubation time are not independent (Figure 4). The highest dry matter content was recorded in the 1_MTG_0T sample (32.53%), while the lowest was observed in the 0_MTG_3T variant (29.61%). A similar pattern was observed for the dry matter of defatted mass, with a significant interaction effect (*p* < 0.0001) and significant individual effects for both MTG concentration and incubation time. Regarding fat content, the main effects and interaction between MTG concentration and incubation time were also significant (*p* < 0.0001), highlighting the importance of both factors in influencing all mentioned quality parameters in meat analogs. These results indicate that higher MTG concentrations promote a more compact protein structure that retains moisture more effectively, reducing evaporation during processing, contributing to increased dry matter retention [3]. Microbial transglutaminase modifies the hydrophobicity and hydrophilicity of the protein surface, affecting interactions with fat molecules. As MTG concentration increases, the protein matrix exhibits greater hydrophilicity, which enhances water retention while reducing fat adhesion. Consequently, this shift in molecular interactions discourages fat incorporation into the structure, contributing to lower fat content [41].

### 3.4. Total Water and Fat Leakage, the Water and the Fat Content in the Total Leakage

**Figure 5:**
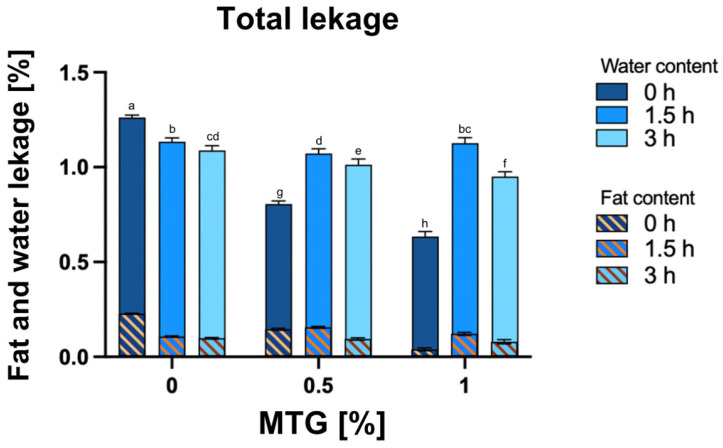
Effect of MTG concentration and incubation time on the total water and fat leakage, including the water content and fat content in the overall leakage. The data represent the mean and the error bars represent the standard deviation (*n* = 6). ^a–h^ means statistically significant differences (*p* ≤ 0.05).

Figure 5 presents the results for the total water and fat leakage of the samples. The effects of varying concentrations of MTG and incubation times on total water and fat leakage revealed significant interactions between the tested factors. Both MTG concentration and incubation time significantly influenced total leakage, with *p*-values less than 0.0001 for all variations. Specifically, the interaction effect was highly significant (*p* < 0.0001), indicating that the combined influence of MTG concentration and incubation time is critical for understanding their impact on leakage properties. The highest leakage was observed in the 0_MTG_0T sample (mean 1.262) and the lowest in 1_MTG_0T one (mean 0.634).

For both the water content and fat content in total leakage, significant differences were observed under different conditions, with the main and interaction effects being significant (*p* < 0.0001). The analysis showed that increasing the MTG concentration significantly reduced the water content of the leakage, while the fat content exhibited a contrasting trend. Improved protein structure facilitated by higher MTG concentrations enhances water-binding capacity and moisture retention, resulting in reduced total leakage. Additionally, with higher MTG concentrations and extended incubation times, the fat content of the leakage decreased. This trend indicates that a higher MTG concentration strengthens the protein network, effectively reducing leakage, although prolonged incubation may begin to affect stability [42,43].

### 3.5. Thermal Loss

**Figure 6:**
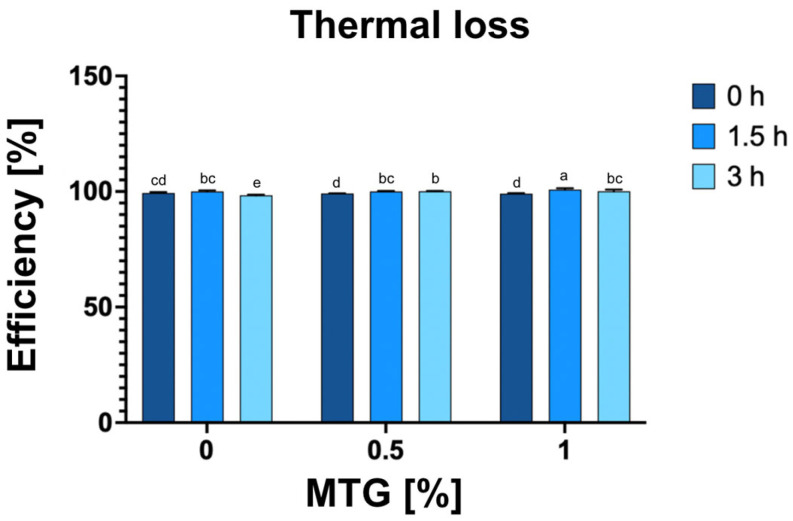
Effect of MTG concentrations over incubation times on percentage process efficiency of meat analog. The data represent the mean and the error bars represent the standard deviation (*n* = 6). ^a–e^ means statistically significant differences (*p* ≤ 0.05).

The analysis of thermal loss in relation to MTG concentration and incubation time revealed significant effects of both factors on the observed results (Figure 6). The thermal efficiency of the meat analog ranged from 98.37% for the 0_MTG_3T variant to 100.9% for the 1_MTG_1.5T variant (Figure 5). The results indicated that the incubation time had the greatest impact, contributing 35.07% to the observed variation. This suggests that longer incubation periods allow for more extensive protein denaturation and moisture loss, which are critical factors influencing the thermal properties of meat analogs. Higher MTG concentrations enhance textural properties and moisture retention, while prolonged incubation promotes protein interactions that contribute to greater thermal stability [44,45]. Therefore, optimizing MTG concentration and incubation time is essential for improving the thermal stability and overall efficiency of meat analog production.

### 3.6. Textural Properties of Samples

**Figure 7:**
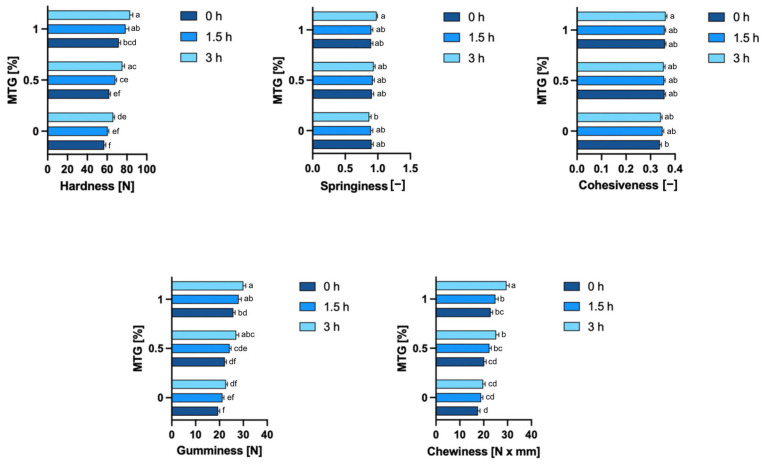
Textural properties of meat analogs with varying MTG concentrations and incubation times. The data represent the mean and the error bars represent the standard deviation (*n* = 38). ^a–f^ means statistically significant differences (*p* ≤ 0.05).

As shown in Figure 7, MTG concentration significantly influenced all textural properties of meat analogs except springiness. Incubation time also had a notable effect, primarily increasing hardness, gumminess, and chewiness as cross-linking continued over time. Analyzing the effects of MTG concentration and the incubation time, the 0_MTG_0T sample exhibited the lowest hardness (57.08 N), while the highest hardness was observed in the 1_MTG_3T variant (83.14 N). Increasing the MTG concentration resulted in higher hardness values, indicating a firmer structure as the enzyme cross-linked more protein molecules, forming a denser matrix. The incubation time also contributed to increased hardness, suggesting that the enzyme continued to strengthen the protein network with prolonged exposure. These findings align with previous studies demonstrating the role of MTG in enhancing textural firmness through protein cross-linking. Hui and Xing [46], Zheng [47] and Rohithkumar et al. [48] similarly reported that higher transglutaminase levels and longer incubation increased hardness in tofu and related products. Wen et al. [49] found that increasing MTG concentrations accelerated the enzymatic reaction of protein cross-linking, generating a stronger internal support within protein gels and increasing resistance to external damage.

Unlike hardness, springiness was not significantly influenced by either MTG concentration or incubation time (*p*-values of 0.1395 and 0.3762, respectively). Springiness, which reflects the resilience of the product after deformation, remained relatively stable across all tested conditions with values ranging from 0.8691 (0_MTG_3T) to 0.98 (1_MTG_3T). In terms of cohesiveness, the lowest value was recorded in sample 0_MTG_0T (0.34), while the highest was observed in 1_MTG_3T variant (0.3616). Cohesiveness was significantly influenced by MTG concentration (*p* = 0.0001), but incubation time did not have a significant effect (*p* = 0.7868). The interaction effect for cohesiveness was also non-significant (*p* = 0.6147), indicating that while increased MTG concentration improves cohesiveness, the duration of incubation does not contribute to further changes. This is consistent with the findings of Amjanyakun et al. [50], who observed limited effects of transglutaminase on springiness and cohesiveness in plant-based meat analogs. This suggests that while transglutaminase promotes protein cross-linking, the resulting structural enhancements primarily contribute to firmness rather than elasticity.

Gumminess and chewiness are critical textural parameters that reflect the overall structural integrity and resilience of a product under compression and during chewing, respectively. Both parameters were significantly affected by MTG concentration (*p* < 0.0001) and incubation time (*p* < 0.0001), while their interaction was non-significant, indicating that the effects of MTG concentration and incubation time operate independently. For gumminess, increasing MTG concentration led to a pronounced increase in values, with the highest gumminess observed in sample 1_MTG_3T (30.00 N), while the lowest was found in sample 0_MTG_0T (19.40 N). Chewiness followed a similar pattern to gumminess, with higher MTG concentrations and longer incubation times both contributing to increased values. The maximum chewiness was observed in 1_MTG_0T variant (29.58 N × mm), and the lowest chewiness occurred in 0_MTG_0T (17.60 N × mm). The researchers confirm these results by showing that different enzyme concentrations can affect the textural properties of proteins, reinforcing the view that enzymatic cross-linking is essential to achieve the desired textural results [44,51]. This supports the idea that transglutaminase enhances protein cross-linking, contributing to a denser, more consistent structure that resists deformation under compression, indicating that MTG plays a crucial role in modifying the textural attributes of meat analogs, making them more meat-like in consistency and mouthfeel [52,53].

## 4. Conclusions

Overall, this study highlights the pivotal role of microbial transglutaminase (MTG) in enhancing the quality of chicken meat sausage analogs. Higher MTG concentrations and extended incubation times significantly improved protein content, hardness, cohesiveness, gumminess, and chewiness, thereby enhancing the mechanical strength and mouthfeel of meat analogs. MTG achieves these textural improvements by forming a three-dimensional protein network that supports structural integrity while enhancing moisture retention. Variants with 1% MTG concentration consistently displayed optimal properties, with the 1_MTG_3T variant showing the highest protein content and rheological performance. These findings underscore the potential of MTG to improve product quality across formulations. As demand for meat substitutes grows, MTG-enriched meat analog formulations represent a promising innovation pathway within this expanding sector. Future research on this protein texturization process using transglutaminase to produce a meat-like texture and structure will include microscopic morphology and sensory analysis.

## Figures and Tables

**Table 1:** Experimental design.

Variants Coding	Variability Factors	
	Microbial Transglutaminase MTG [%]	Incubation Time T [h]
0_MTG_0T	0	0
0_MTG_1.5T	0	1.5
0_MTG_3T	0	3
0.5_MTG_0T	0.5	0
0.5_MTG_1.5T	0.5	1.5
0.5_MTG_3T	0.5	3
1_MTG_0T	1	0
1_MTG_1.5T	1	1.5
1_MTG_3T	1	3

## Data Availability

The original contributions presented in the study are included in the article; further inquiries can be directed to the corresponding author.
